# Should We Offer In Vitro Fertilization to Couples with Unexplained Recurrent Pregnancy Loss?

**DOI:** 10.3390/jcm8112001

**Published:** 2019-11-16

**Authors:** Michal Kirshenbaum, Raoul Orvieto

**Affiliations:** 1 Department of Obstetrics and Gynecology, Chaim Sheba Medical Center, Tel-Hashomer, Ramat Gan 52621, Israel; michal.siegel@gmail.com; 2 Sackler Faculty of Medicine, Tel Aviv University, Tel Aviv 39040, Israel; 3 The Tarnesby-Tarnowski Chair for Family Planning and Fertility Regulation, Sackler Faculty of Medicine, Tel-Aviv University, Tel Aviv 39040, Israel

**Keywords:** repeated pregnancy loss, IVF, ART, adds-on, outcome

## Abstract

In clinical practice, empirical treatments are often offered to couples with recurrent pregnancy loss, including in vitro fertilization. Given that most patients with recurrent pregnancy loss are fertile, the scientific rationale of in vitro fertilization for these couple is debatable. This review will discuss the potential benefits of using in vitro fertilization in couples with recurrent pregnancy loss, such as shortening the time to conceive, optimizing the timing of conception, improving gamete and embryo quality, endometrial receptivity and the use of “adds-on”. At present, there is not enough evidence to justify IVF as a treatment option in couples with unexplained recurrent pregnancy loss.

## 1. Introduction

Recurrent pregnancy loss (RPL) is defined as two or more miscarriages, not necessarily consecutive, and represents a significant health problem [1,2]. The prevalence of RPL is approximately 5% [2]. Potential causes of RPL are parental carriers of structural chromosome rearrangement, uterine anomalies, endocrine disturbances and antiphospholipid antibodies. Nonetheless, even after comprehensive evaluation, a cause for RPL is identified in less than 50% of couples [3]. In RPL, when no underlying cause can be identified, empirical treatments are often offered, including assisted reproductive technique (ART). Moreover, although most patients with RPL do not have fertility problems, they are commonly treated by in-vitro fertilization (IVF) with a selection of adjunct treatments or ‘add-ons’, aiming to improve their chance of a live birth. Other so called benefits are quoted to the patient such as lessening the time to conceive, improving embryo quality, improvement of implantation, and improved synchrony between endometrium and embryo. In this review, we will present the clinical evidence and assess the benefit or lack of benefit of the various options offered in ART to unexplained RPL patients. 

## 2. Subsequent Live Birth Rate

In unexplained RPL, the subsequent live birth rate is known to be dependent on certain factors. Briefly, these include the number of previous live births, maternal age, genetic aberrations in the previous pregnancy and whether the losses are early or late. Therefore, if IVF is used, the subsequent live birth rate should be matched for all these prognostic confounders. A thorough literature search could not reveal any publications on subsequent live birth rates of RPL patients undergoing IVF, that matched patients according to the number of previous miscarriages, or any of the aforementioned prognostic confounders. 

Another problem in assessing the live birth rate after IVF in RPL, is how to quote the live birth rate. In RPL, the live birth rate is quoted as the number of live births per pregnancy. In IVF the clinical pregnancy rate is often used as a measure of the treatment success per started cycle. In RPL, the clinical pregnancy rate is meaningless, as RPL patients generally conceive easily. Even if the rate is quoted as live births per cycle, the time lost in preparing for IVF will not be taken into account. If IVF is not used, the patient may well conceive in the interval while planning for IVF. 

Perfetto et al. [4] has quoted the subsequent live birth rate in 98 patients who conceived spontaneously compared to 68 patients undergoing infertility treatment including ovulation induction, intra-uterine insemination, or IVF, and a third group undergoing preimplantation genetic testing for aneuploidy (PGT-A). Eighty-eight percent of patients conceived within six months in the spontaneous pregnancy group compared to 84% in the fertility treatment group and 70% in the PGT-A group. The live birth rates were similar in all groups 77% and 73% and 70%, respectively (*p* = 1.0). The clinical miscarriage rate and the biochemical pregnancy rate were also similar between the three groups: 18 and 6% in the spontaneous conception group, 16 and 11% in the IUI/IVF group and 13 and 9% in the PGT-A group, respectively. However, it must be borne in mind that up to 33% of patients with RPL do have periods when they fail to conceive [5]. Some of these patients will require IVF, but the IVF will be for failure to conceive rather than RPL. 

Hence, there is no data to support using empirical fertility treatment including IVF to improve the live birth rate in RPL.

## 3. Time to Conceive

Previous studies have reported a longer mean interval to subsequent conception after a pregnancy loss, compared to the time to conceive before a pregnancy loss [6,7]. The emotional impact of RPL and the strong desire to conceive, as early as possible, have led patients and physicians to consider fertility treatment, aiming at reducing the time interval to the next pregnancy. Kaandorp et al. [8] assessed the time to conception in 251 women with unexplained RPL. In their study, time to conception was calculated from the diagnosis of unexplained RPL until the first day of the menstrual cycle in which conception occurred. The mean patients’ age was 34 ± 5 years, the median number of preceding miscarriages was three (range 2–15), with a median gestational age of eight weeks (range 6–17). Thirteen percent of the study patients conceived with ART; although no separate analysis was performed for this group. The cumulative incidence of conception was 56% after six months, 74% after 12 months and 86% after 24 months, of which 65% resulted in a live birth. The median time to subsequent conception was 21 weeks (inter quartile range of 8–55). According to the literature, cycle fecundity in normal fertile couples is 20–30% and the cumulative fecundity is 85% and 93% after one or two years, respectively [9,10]. Given that the mean patients’ age in the study by Kaandorp et al. [6] was 34 years, the cumulative pregnancy rate observed in this study is similar to that reported for the general population. 

As was already shown above, Perffeto et al. compared the time to pregnancy, as well as the miscarriage rate and subsequent live birth in fertile patients with RPL, who attempted to conceive spontaneously, and those that opted to undergo fertility treatment [4]. In their study, 190 patients with two or more clinical miscarriages were followed for a subsequent pregnancy for a minimum six months, beginning after a complete work up investigation of RPL. Among the 98 patients who conceived spontaneously, the median time to pregnancy was two months (range 1–10) and 88% conceived within six months. The median time to pregnancy among the 68 women who conceived with fertility treatment was significantly longer: three months (range 1–9) for controlled ovarian stimulation with intra-uterine insemination (IUI), four months (range 1–12) with IVF and give months (range 2–10) for PGT-A. In patients achieving pregnancy with fertility treatment, excluding PGT-A, 84% conceived within six months. For patients conceiving with PGT-A, the time to conceive was significantly longer. Only 70% conceived within six months. The authors concluded that in young fertile patients with RPL, there does not appear to be a clinical benefit to using fertility treatment in order to reduce the time to subsequent pregnancy. Of notice, two differences between the Perffeto et al. study groups might influence the study results and conclusions. The patients that attempted to conceive spontaneously were slightly younger than the patients undergoing fertility treatment (34.5 vs. 35.6) and the subset of women who used PGT-A were even older, with a mean age of 36.7 years. Although this difference was not statistically significant (*p* = 0.12), it might have affected the time to pregnancy, as the conception rate declines with advanced maternal age [11,12]. Moreover, women in the fertility treatment group had a significantly longer median time to conceive in prior pregnancies (3 vs. 2 months). While time to pregnancy seems to be similar across successive pregnancy attempts [13], it is possible that the difference in time to pregnancy between the groups was due to a different fertility potential. 

Murugappan et al. retrospectively compared the outcomes among patients with RPL intending to pursue PGT-A and patients who were managed expectantly and attempted spontaneous conception for an interval of six months [14]. All cycles of PGT-A were included, including cancelled cycles and those that did not lead to embryo transfer. The median time to pregnancy was longer in the PGT-A group (6.5 months) than that of the spontaneous conception group (3 months). Murugappan et al. concluded that PGT-A should not be offered for patients who feel an urgency to conceive.

In fertile couples with unexplained RPL, it seems that there is no benefit in fertility treatment, including IVF, in order to shorten the time to the next pregnancy. 

## 4. Sperm Selection Techniques to Improve Sperm Quality 

Until recently, the assessment and treatment of couples with RPL has been directed exclusively to investigation of the female rather than the male partner. The herewith discussion will be limited to the evidence that sperm selection can improve outcome when empirical IVF is used for RPL. Of notice, a recent Cochrane review revealed uncertainty regarding the effect of sperm selection techniques on live birth, miscarriage, or pregnancy [15].

It has been reported that the sperm of men with RPL have significantly reduced viability, with an increased proportion of DNA damage when compared with fertile controls [16,17,18]. Sperm function parameters such as hypo-osmotic swelling, acrosomal status and nuclear chromatin decondensation have also been found to be reduced in the male partners of RPL couples compared to fertile males [19]. In conventional IVF, various sperm preparation techniques have been used, aiming to improve the fertility potential. These techniques include semen washing, density gradient centrifugation, swim up technique and electrophoretic sperm selection, followed by IVF. Another method for sperm improvement selects non-apoptotic sperm, based on the presence of phosphatidylserine on the external surface of the sperm membrane in the early stages of apoptosis. Magnetic activated cell sorting and glass wool separation columns utilize the magnetic properties of phosphatidylserine to separate apoptotic sperm from non-apoptotic sperm. However, none of these techniques have been reported to improve the live birth rate in RPL.

Physiological intracytoplasmic sperm injection (PICSI) utilizes the presence of hyaluronic acid (HA) binding sites on the sperm plasma membrane. HA binding sites indicate sperm maturity, and its ability to attach the extracellular matrix of the cumulus oophorus. A recent Cochrane review aiming to evaluate the impact of advanced sperm selection techniques on ART outcomes could not find sufficient evidences to allow the review authors to determine whether sperm selected by surface charge, sperm apoptosis or hyaluronic acid binding have any additive value over conventional selection [20]. No difference was found between the methods in terms of live births, clinical pregnancy or miscarriage rates. However, this meta-analysis was not restricted to RPL patients. A recent RCT [21], investigating the efficacy of PICSI versus standard ICSI for improving livebirth rates among couples undergoing fertility treatment could not demonstrate any significant advantage of PICSI over ICSI and they therefore concluded that at present, the wider use of PICSI is not recommended. 

Intracytoplasmic morphologically selected sperm injection (IMSI) is another technique to select sperm for injection to the egg by examining the sperm organelles morphology, such as the acrosome, postascrosomal lamina, neck, mitochondria, tail and nucleus (MSOME) using ultra-high magnification (≥6000×) microscopy. Although initial reports have shown that IMSI is associated with a higher pregnancy rate and lower miscarriage rate [22,23], both the effectiveness and safety of IMSI in clinical practice remain unclear. A Cochrane review has found an increased clinical pregnancy rate using IMSI compared to ICSI, although, there was no difference regarding the live birth rate or the miscarriage rate [24]. 

In conclusion, although sperm selection techniques might improve sperm quality and overcome potential male subfertility in RPL couples, the evidence is insufficient to recommend use in RPL. 

## 5. Improved Embryo Quality

Women natural fecundity decreases gradually but significantly beginning approximately at age 32 years and decreases more rapidly after age 37 years. Similar trend has been observed when analyzing data derived from IVF cycles. This age-related decline in fertility is accompanied by significant increases in the rates of aneuploidy and spontaneous abortion [25]. 

### 5.1. Embryo Morphology

Embryo morphology is thought to be highly indicative of pregnancy outcome and therefore morphological grading of the embryo may allow the selection of “the best” embryos for transfer. Over the past decade, with the development of sequential culture media, there has been a steady shift in practice to transfer day five or six embryos, at the blastocysts stage. The argument for blastocyst transfer is that the blastocyst has undergone a self-selection process, in which only the most viable embryos have survived and developed. A Cochrane review demonstrated a higher clinical pregnancy and live birth rates in fresh blastocyst transfer compared to fresh cleavage stage embryo transfer. [26]. Again the meta-analysis was performed for infertile rather than RPL patients. 

A large proportion of morphologically normal day three embryos are chromosomally abnormal. Aneuploid embryos on day three often fail to reach the blastocyst stage [27,28]. Several studies have investigated the relationship between morphology, euploidy and the implantation rate of cleavage stage and blastocyst stage embryos. Majumdar et al., in their retrospective analysis, have demonstrated that blastocyst morphology and the rate of development were significantly associated with euploidy, whereas cleavage stage morphology was not. Nonetheless, implantation rates were similar for all transferred euploid blastocysts, irrespective of their morphology or their rate of development [28]. Similarly, Capalbo et al. found a correlation between blastocyst morphology and euploidy, although the implantation potential of euploid embryos was similar despite different morphologies and development rates [29]. The association between blastocyst morphology and aneuploidy explains the higher implantation potential of good quality embryos reported during conventional IVF cycles. However, the commonly used parameters of blastocyst evaluation are not good indicators to improve the selection of euploid embryos. 

In conclusion, when IVF treatment is used for selecting high quality embryos, blastocyst morphology can be used to slightly reduce the risk of transferring aneuploid embryos. Nonetheless, in the absence of studies evaluating this potential advantage in women with RPL, we cannot recommend its use.

### 5.2. Time-Lapse System Embryoscopy

Traditionally, embryo assessment has involved removing embryos from a conventional incubator for quality assessment by an embryologist, under a light microscope. Recently, time-lapse systems (TLS) have been developed which can take digital images of embryos at frequent time intervals. Hence, the quality of the embryos can be assessed without physical removal from the incubator. The potential advantages of TLS include maintenance of a stable culture environment, limiting the exposure of embryos to changes in gas composition, temperature and movement. TLS has the potential advantage of improving embryo selection for ART treatment by utilizing additional information gained through continuously monitoring embryo development.

Although the clinical value of TLS has been validated in some studies [30,31], literature reviews have provided controversial data, leading to ongoing debate. A Cochrane review and a recent meta-analysis studied the advantage of TLS embryonal assessment versus conventional embryonal incubation and assessment [32,33]. No difference was found between the two interventions in terms of live birth or miscarriage rates. Another review, evaluated the association between morphokinetic parameters and embryo ploidy to evaluate whether TLS can replace PGT-A [34]. No single or combined morphokinetic parameter was consistently identified as predictive of embryonic euploidy. 

Currently there is insufficient evidence that TLS is superior to conventional methods for human embryo incubation and selection or prediction of embryonic euploidy. Although maximizing embryonal quality might improve pregnancy outcome in couples with RPL, TLS during IVF cannot currently be recommended. 

### 5.3. Preimplantation Genetic Testing-Aneuploidy (PGT-A) 

A large majority of early pregnancy losses are the consequence of chromosomal abnormalities of the conceptus [2]. If performed correctly, genetic analysis of products of conception, therefore, offers important information about potential causes of pregnancy loss and assists in the planning of appropriate investigations and treatment. PGT-A currently utilizes trophectoderm biopsy and next-generation sequencing (NGS) in an attempt to detect embryonic aneuploidy in a trophectoderm biopsy obtained at the blastocyst-stage. The current version of PGT-A is claimed to have significantly improved our ability to accurately diagnose embryonic aneuploidies without compromising the embryo’s implantation potential. 

Within this context, the European Society of Human Reproduction and Embryology (ESHRE) recently published a new guideline on RPL [2], in which PGT for monogenic/single gene defects (PGT-M) or chromosomal structural rearrangements (PGT-SR) were described as established alternatives to invasive prenatal diagnosis and might avoid pregnancy termination in couples with a high risk of transmitting genetic disorders. Importantly, the ESHRE guideline made no recommendation for any form of PGT in couples with unexplained RPL without known chromosomal abnormalities. PGT-A, therefore, is not indicated in couples with unexplained RPL according to the ESHRE guideline.

Starting in 2015, the clinical utility of PGT-A faced increasing scrutiny [35], and cases reporting on patients experiencing spontaneous miscarriages after PGT-A, in which chromosomal reassessment was found to be aneuploid, raising the specter of false-negative TEBs [36]. At the same time, concerns about false-positive TEBs arose in relative good prognosis patients, who repeatedly underwent IVF cycles without ever reaching embryo transfers because all embryos were reported as aneuploid. Suspicion that false-positive embryos were erroneously labeled as aneuploid, led to the transfer of such embryos, resulting in a surprisingly high number of normal live birth and a surprisingly low miscarriage rates [37]. Further evidence for inaccurate diagnoses in cases of TE mosaicism came from studies of multiple TEB biopsies in the same embryos, demonstrating up to 50% divergence between biopsies of the same embryos in the same laboratories, and up to approximately 80% divergence between multiple biopsies in different laboratories [37,38,39,40]. Moreover, while several studies claimed improved clinical IVF outcomes following PGT-A [41], others scrutinized those studies as being severely biased, uniformly reporting IVF outcomes only with regard to embryo transfers in first fresh IVF cycles, instead of looking at outcome which should be based on intention to treat analyses and includes total reproductive potential for each initiated IVF cycle. Of interest are the results of a recent RCT, which included patients age 25–40 years with at least two blastocysts of sufficient quality for biopsy and vitrification (again, patients were not randomized per started cycle), randomized to PGT-A or morphology alone. In this study, ongoing pregnancy rate was equivalent between the two arms, with no significant difference per embryo transfer (50% vs. 46%) or per intention to treat at randomization (41.8% vs. 43.5%). Moreover, miscarriage rates per transfer were 10%, regardless of maternal age in both the PGT-A arm and the control arm [42]. 

Moreover, due to the lack of properly conducted prospective clinical trials, a theoretical model was published for PGT-A. The theoretical model, relied on evidence- based data in the literature on blastulation and aneuploidy rates, the rate of mosaicism, technical errors and implantation/live birth rates of PGT-A and non- PGT-A cycles at cleavage and blastocyst stage [43]. The model clearly revealed the highest LBRs in patients not undergoing PGT-A (21.4–50%), while patients undergoing PGT-A blastocysts transfers achieved the lowest LBR (7.6–12.6%).

In a systematic review on PGT-A for unexplained RPL patients, Musters et al. [44] concluded that there is no improvement in the live birth rate with PGT-A. Of notice, the included studies were of small sample sizes, with different end-points, and used FISH. In a recently published study, comparing PGT-A and expectant management (EM), Murugappan et al. [14] reported similar pregnancy, live birth and clinical miscarriage rates at PGT-A or expectant management, with a shorter time to pregnancy (3.0 vs. 6.5 months) in the patients managed expectantly. Moreover, Murugappan et al. did not find PGT-A a cost-effective strategy for increasing live births [45]. 

Based on the above observations, the previously referred to new ESHRE guideline [2] made no recommendation with regard to the use of PGT-A in patients with unexplained RPL. The ESHRE guideline mentioned the study by Shahine et al. [46], reporting that in couples with unexplained RPL with diminished ovarian reserve, there was a higher percentage of aneuploidy in blastocysts, and more initiated IVF cycles with no embryo transfers. Hence, Shahine et al.’s study clearly contradicts the use of PGT-A in unexplained RPL. 

To date, not a single study in the literature has suggested improved live-birth rates in RPL patients after PGT-A. Moreover, it appears increasingly obvious, that the basic biology of the preimplantation human embryo, simply, does not support the PGS-hypothesis. It is therefore becoming increasingly difficult to expect any benefit from PGT-A.

## 6. Improving Implantation

### 6.1. Assisted Hatching 

Assisted hatching (AH) is a manipulation of the zona pellucida in order to facilitate implantation. AH involves thinning the coat surrounding a fertilized egg, or making a hole in it. A variety of techniques have been employed to assist embryo hatching, including partial mechanical zona dissection, zona drilling and zona thinning, making use of acid tyrodes, proteinases, piezon vibrator manipulators and lasers [47]. Harper et al. reviewed the literature evaluating the effect of AH on IVF treatment and concluded that it increases clinical pregnancy and multiple pregnancy rates, but not live birth rate [31].

Since no single study has been able to demonstrate sufficient evidence of a benefit in the live birth rate of AH in RPL, we cannot recommend use. 

### 6.2. Biologic Glue

In an attempt to increase the success rate of IVF, various compounds have been added to the embryo transfer medium in order to improve adherence and subsequent implantation and pregnancy rates. Glycoprotein hyaluronan (HA) forms a viscous solution, that might enhance the embryo transfer process and prohibit expulsion, or it may facilitate diffusion and integration of the embryos in the viscous solution that characterizes intrauterine secreted fluid [48]. The contribution of HA to implantation may also be receptor mediated, as the primary receptor for HA is CD44, which is expressed both on the preimplantation embryo and on the endometrial stroma [49]. A Cochrane review of 17 randomized control trials (RCT), aiming to evaluate the supplementation of HA to embryo transfer medium, demonstrated an improvement in clinical pregnancy and live birth rates, with an associated increase in the multiple pregnancy rate [50]. However, a more recent RCT found no significant difference in clinical pregnancy, implantation or delivery rate between the HA group and the control group [51]. 

The use of HA in RPL couples might potentially improve implantation and ongoing pregnancy rate. However, before conclusion can be drawn, RCTs are needed to evaluate efficacy in RPL patients.

## 7. The Window of Implantation-Improving Synchronization

Timing of conception in relation to ovulation may affect the spontaneous miscarriage rate. Previous studies have suggested that prolonged exposure of the gametes to the female reproductive tract may have a devastating effect on the ongoing pregnancy rate. Furthermore, aging of both spermatozoa and ova before fertilization was accompanied by a higher probability of miscarriage [52,53]. Gray et al. assessed the effect of timing of conception on the risk of miscarriage in women conceiving naturally [54]. Conception on the day of ovulation or the day preceding ovulation was considered optimal. Among patients who had miscarried in a prior pregnancy, the incidence of miscarriage was significantly higher in the index pregnancy with non-optimally timed conceptions (22.6%), as compared with optimally timed conceptions (7.3%). This association was not observed among women with no history of pregnancy loss. Likewise, studies that assessed the optimal time of conception among women with no history of miscarriage reported no increased risk of miscarriage following conception remote from the day of ovulation [55,56]. The authors postulated that some couples are predisposed to genetic abnormalities in the gametes if fertilization does not occur at the optimal time of the cycle. 

Synchronization between embryonic development and endometrial decidualization is essential for adequate implantation. The window of implantation (WOI) is a temporally restricted phase that is multifactorial, during which changes occur at the molecular, cellular and tissue levels. It is assumed that the endometrial WOI begins on cycle days 19 or 20 of an idealized 28 days cycle and lasts for 4 to 5 days [57]. Wilcox et al. studied the relation between the time of implantation and the outcome of pregnancy in couples with no history of fertility problem trying to naturally conceive [58]. They found that in most successful human pregnancies, the conceptus implanted 8 to 10 days after ovulation and later implantation, i.e., beyond the normal period of endometrial implantation, is strongly associated with increased early pregnancy loss. 

Noyes et al., were the first to assess the uterine receptivity timeline and defined a series of morphological criteria, to date the endometrium [59]. RPL has been reported to be associated with retarded endometrial development in the peri-implantation period, known as the luteal phase defect (LPD). A maturation delay has been described in 17–28% of patients with RPL [60,61]. RPL has also been associated with abnormal endometrial expression of various mediators and metabolic factors in the secretory and peri-implantation phases [62,63]. 

ART might be justified in patients with RPL, in order to avoid non-optimal timing of intercourse or conception. Moreover, assessing the endometrium in the cycle prior to embryo transfer might enhance synchronization and evaluate the quality of endometrial receptivity. In the past, Noyes et al.’s histologic criteria have been the gold standard for evaluating endometrial development and receptivity. However, histologic dating is prone to intra- and inter-observer variability and tissue fixation artefacts. In a search for an accurate method to evaluate endometrial receptivity, many structural characteristics and molecules have been studied, including ultrasonographic measurement of endometrial thickness, structural examination by electron microscopy, immunological markers, steroid hormones and receptors and protein expression profiles [57,64]. A new approach to assess endometrial function is the endometrial receptivity array (ERA) test, based on analysis of expression of 238 genes that are found to be involved in the receptivity of the endometrium [65]. The value of the ERA test is controversial with some studies supporting the utility and accuracy of the ERA test and some have not [66,67,68,69]. However, no report has assessed its role in patients with RPL. 

## 8. Conclusions

Although subfertility is not a problem in most couples with RPL, ART is often advised in RPL couples. However, scientific evidence is lacking. Patients might be interested in IVF in order to shorten time to conceive, but to date, IVF has not shown any benefit regarding the time to conceive. 

Embryo quality has a significant role in the success of an ART cycle. ART includes methods to improve gametes and embryo quality, such as sperm selection, PGT-A and morphologic evaluation. Although maximizing embryonal quality might improve the pregnancy outcome in couples with RPL, further adequately powered studies are needed to assess the results.

An abnormal endometrial microenvironment and changes in the functional expression of endometrial genes and protein might contribute to an abnormal embryonal-maternal interaction, resulting in pregnancy failure. Endometrial sampling for assessing endometrial receptivity and accurately timed embryonal transfer might improve this embryonal-maternal interaction. Nonetheless, due to the lack of studies investigating these methods in RPL patients, IVF cannot be recommended for this purpose. 

Furthermore, several “adds on” to IVF treatment, including assisted hatching, biologic glue and immunologic therapy have also been suggested to improve implantation and live birth rates. Since their efficacy is controversial, these cannot be currently recommended. 

In conclusion, ART, without secondary subfertility, cannot be supported as a treatment intervention for couples with unexplained RPL, because of the lack of adequate clinical studies. Further research and review into the underlying etiology of RPL, including studies assessing IVF success in RPL patients are required. These may help to clarify the proper approach to RPL patients and to aid fertility specialists and their patients in the decision-making process.
